# *MiR-10a*, *27a*, *34b/c*, and *300* Polymorphisms are Associated with Ischemic Stroke Susceptibility and Post-Stroke Mortality

**DOI:** 10.3390/life10120309

**Published:** 2020-11-25

**Authors:** Chang Soo Ryu, Seung Hun Oh, Kee Ook Lee, Han Sung Park, Hui Jeong An, Jeong Yong Lee, Eun Ju Ko, Hyeon Woo Park, Ok Joon Kim, Nam Keun Kim

**Affiliations:** 1 Department of Biomedical Science, College of Life Science, CHA University, Seongnam 13488, Korea; regis2040@nate.com (C.S.R.); hahnsung@naver.com (H.S.P.); tody2209@naver.com (H.J.A.); smilee3625@naver.com (J.Y.L.); ejko05@naver.com (E.J.K.); aabb1114@naver.com (H.W.P.); 2 Department of Neurology, CHA Bundang Medical Center, School of Medicine, CHA University, Seongnam 13496, Korea; ohsh72@chamc.co.kr (S.H.O.); niceiatros@cha.ac.kr (K.O.L.)

**Keywords:** ischemic stroke, microRNA, polymorphism, survival, diagnosis

## Abstract

A recent study of the ischemic stroke described the roles played by miRNAs in the downregulation of specific cell-cycle gene expression and it is thought to require the development of biomarkers for the prognostic of ischemic stroke. Here, we hypothesized that four miRNA polymorphisms (*miR-10a*, *miR-27a*, *miR-34b*/c, and *miR-300*) may affect stroke susceptibility and mortality. Blood samples were collected from 530 patients and 403 controls. Genetic polymorphisms were detected by polymerase chain reaction (PCR)-restriction fragment length polymorphism analysis and real-time PCR. We found that the *miR-300* rs12894467 TC genotype and the dominant model (AOR: 2.069, *p*-value: 0.017; AOR: 1.931, *p*-value: 0.027) were significantly associated with an increased risk for the ischemic stroke subtype. In Cox proportional hazard regression models, the *miR-10a* rs3809783 A>T and *miR-34b/c* rs4938723 T>C polymorphisms were associated with the mortality rates among ischemic stroke patients. We found that a *miR-300* polymorphism was associated with increased ischemic stroke susceptibility among the Korean population. Additionally, polymorphisms in *miR-10a* and *miR-34b/c* were associated with the increased or decreased mortality of ischemic stroke patients. This study marks the first report of an association between ischemic stroke and miRNA polymorphisms (*miR-10a*A>T, *miR*-*27a*T>C, *miR-34b/c*T>C, and *miR-300*T>C) in the Korean population.

## 1. Introduction

Ischemic stroke is caused by several gene–gene and gene–environment interactions [1,2] and can be affected by genetic and environmental factors, including advanced age, diabetes mellitus (DM), family or personal history of stroke, high cholesterol, smoking, hypertension (HTN), hyperlipidemia, and metabolic syndrome (MetS), among which DM, HTN, smoking, and hyperlipidemia are considered to be the primary contributors [3]. One of the primary challenges associated with apposite stroke management is the validation of significant biomarkers for the diagnosis and therapeutic observation of stroke patients [4,5]. However, existing biomarkers have limitations, requiring the development of additional biomarkers for diagnostic and prognostic predictions [6,7]. Among miRNA-associated studies in several conditions, miRNAs have been reported to act as biological regulators of cell-cycle progression and neurodegeneration [8,9,10,11,12]. A recent study described the roles played by miRNAs in the downregulation of specific cell-cycle gene expression [13]. 

MiRNAs can bind with several sites in the 3′-untranslated regions (3′-UTRs) of mRNAs, affecting their abilities to be translated [14]. Many previous studies have demonstrated that the expression levels of target genes can be regulated by several miRNAs [15,16,17], and miRNAs have been examined in several diseases, including stroke [18,19,20,21,22,23]. These studies have provided evidence for the functions of miRNAs and have indicated that miRNA biosynthesis may contribute to important physiological and pathological processes [24,25]. As miRNA studies have progressed, in vivo and human studies have reported the abnormal expression of miRNAs associated with stroke [26,27,28]. These studies have demonstrated that miRNAs may act as key modulators during the pathogenic and pathological events associated with ischemic stroke [29,30,31,32]. Moreover, some studies have suggested that delivered miRNAs could represent a suitable treatment option for stroke patients, further supporting the crucial effects of miRNAs during stroke [33,34]. Consequently, various types of miRNAs have been suggested as potential biomarkers for ischemic stroke, and associations have been demonstrated between miRNA polymorphisms and ischemic stroke occurrence [35,36,37,38,39]. 

Studies of specific miRNAs, including *miR-10a*, *miR-27a*, *miR-34b/c*, and *miR-300*, have demonstrated their abilities to affect the expression of specific target genes associated with thrombosis as well as demonstrating the potential for these miRNAs to regulate the thrombotic signaling pathway during ischemic stroke [14,36,40,41,42,43,44,45,46,47,48,49,50]. Furthermore, *miR-10a* has been shown to regulate platelet-associated gene expression, and plasma levels of this miRNA have also been suggested to act as a potential biomarker for another disease [51,52]. In another study, *miR-27a* was suggested as a potential biomarker or therapeutic target for atrial fibrillation-related stroke [53]. In addition, *miR-300* has previously been demonstrated to be a potential plasma marker in rat models of transient ischemic attack, which shares a similar mechanism with ischemic stroke [42]. Polymorphisms in *miR-10a*, *miR-27a*, *miR-34b/c*, and *miR-300* have also been reported to affect their own expression levels [54,55,56,57,58].

Therefore, we hypothesized that polymorphisms in *miR-10a*, *miR-27a*, *miR-34b*/c, and *miR-300* may affect stroke susceptibility and post-stroke mortality among patients with ischemic stroke, and these polymorphisms have the possibility of affecting miRNA expression. Consequently, we designed a case-control study to investigate the association between four miRNA polymorphisms and ischemic stroke in a Korean population. To the authors’ best knowledge, this study is the first evidence demonstrating a link between these four miRNA polymorphisms and ischemic stroke risk in the Korean population.

## 2. Materials and Methods 

### 2.1. Study Participants

Blood samples were collected from 530 ischemic stroke patients and 403 control participants. All study protocols were reviewed and approved by the Institutional Review Board of CHA Bundang Medical Center in June 2000 (IRB No. 2013–09–073) and followed the recommendations of the Declaration of Helsinki. All patients and controls were recruited from the Department of Neurology of CHA Bundang Medical Center, CHA University, between 2000 and 2008. Written informed consent was obtained from all study participants. Ischemic stroke was defined as a stroke (a clinical syndrome characterized by rapidly developing clinical symptoms and signs of the focal or global loss of brain function) with evidence of cerebral infarction in clinically relevant areas of the brain according to magnetic resonance imaging (MRI) scan finding. Based on clinical symptoms and neuroimaging data, two neurologists categorized whole ischemic strokes into four causative subtypes, using the Trial of Org 10172 in Acute Stroke Treatment (TOAST) criteria [59], as follows: (1) large-artery disease (LAD) was defined by an infarction lesion diameter ≥15 mm, assessed by MRI, and significant (>50%) stenosis of the main brain artery or cerebral cortex artery, assessed by cerebral angiography; (2) small-vessel disease (SVD) was defined by an infarction lesion diameter of <15 mm and ≥5 mm, assessed by MRI, and classic lacunar syndrome, without evidence of cerebral cortical dysfunction or detectable cardiac embolism; (3) cardioembolism (CE) was defined by arterial occlusions and the development of embolus in the heart, as detected by cardiac evaluation; and (4) pathogenesis, which included cases for which the cause of stroke could not be determined and cases that were associated with two or more causes. Based on these criteria, 34.7% (*n* = 184) of stroke patients were categorized as LAD, 28.5% (*n* = 151) were categorized as SVD, 10.6% (*n* = 56) were categorized as CE, and 26.2% (*n* = 139) were categorized as undetermined pathogenesis. In addition, we selected 403 controls that were matched for sex and age within five years in accordance with the patient group. Controls were drawn from subjects visiting our hospitals during the same period for health examinations, including biochemical testing, electrocardiograms, and brain MRIs. Control subjects did not have a recent history of cerebrovascular disease or myocardial infarction. Exclusion criteria were the same as those used for the case group, as mentioned previously. HTN was defined as a systolic pressure of >140 mmHg and a diastolic pressure of >90 mmHg, on more than one occasion, and included patients currently taking hypertensive medications. DM was defined as fasting plasma glucose levels >126 mg/dL (7.0 mmol/L) and included patients currently taking diabetic medications. Smoking referred to patients who were current smokers. Hyperlipidemia was defined as a high fasting serum total cholesterol level (≥240 mg/dL) or a history of antihyperlipidemic agent treatment. Patients were diagnosed with MetS if they possessed more than three of the following five risk factors [60].

### 2.2. Genotyping

Genomic DNA from all participants was extracted from blood leukocytes using a G-DEX^TM^ blood extraction kit (Intron Biotechnology, Seongnam, South Korea) and was performed according to the manufacturer’s instructions. Most of the genetic polymorphisms were confirmed by polymerase chain reaction (PCR) and restriction fragment length polymorphism (RFLP) analyses [61]. One genetic polymorphism was detected by real-time PCR. The *miR-27a* rs895819 T>C, *miR-34b/c* rs4938723 T>C, and *miR-300* rs12894467 T>C polymorphisms were confirmed by the restriction enzyme activities of *Dra*III, *Bcc*I, and *Nsi*1 (New England Bio Laboratories, Ipswich, MA, USA), respectively, at 37 °C for 16–24 hours. The primer sequences of the miRNAs are as follows: *miR-27a* forward sequence “GAA CTT AGC CAC TGT GAA CAC GAC TTC G” and reverse sequence “TTG CTT CCT GTC ACA AAT CAC ATT G,” *miR-34b/c* forward sequence “TCC TCT GGG AAC CTT CTT TGA CCC” and reverse sequence “CTA GTC AAA TAG TGA GCC AGG CAG CT,” *miR-300* forward sequence “AAT AGA TGT GTG ATT CAC CCA CG” and reverse sequence “ATC TAA CAG GTT GCT GGA GTC AG.” Each polymorphism genotype was confirmed by electrophoretic separation on 4% agarose gels. In addition, for each polymorphism, PCR data were randomly selected and the results were verified using DNA sequencing, to validate real-time PCR and RFLP findings.

### 2.3. Post-Stroke Mortality

To estimate the association between these four miRNA polymorphisms and long-term prognosis after ischemic stroke, the time from stroke occurrence to death was recorded. The death dates of each stroke patient (*n* = 530) were confirmed using death certificates from the Korean National Statistical Office. The survival statistics were based on the survival data from 2008 to 2013, and any patients who were still alive on 31 December 2013, were censored.

### 2.4. Statistical Analysis

To compare baseline characteristics between ischemic stroke patients and controls, we used Chi-square tests to analyze categorical data, and the unpaired-samples Student’s *t*-test and Mann–Whitney U test to analyze continuous data. The characteristics were expressed as mean ± standard deviation for continuous variables and number (%) for categorical variables. When comparing continuous variables between ischemic stroke patients and controls, we analyzed them using the unpaired-samples Student’s t-test. During the analysis of continuous variables, the Mann–Whitney U test was performed when the F-test for the equal variances was less than 0.05. When comparing baseline characteristics between ischemic stroke patients and controls, age and gender were not significant, and these results meant no difference between ischemic stroke patients and controls in age and gender. Differences in the identified polymorphism genotype and allele combination frequencies between ischemic stroke patients and control subjects were compared using multivariate logistic regression models and Fisher’s exact test, respectively. The odds ratio (OR), adjusted ORs (AORs), and 95% confidence interval (CI) were utilized to measure the strength of association between various genotypes and stroke. For multivariate analyses, logistic regression analyses were used to adjust for possible confounders, including sex, age, DM, HTN, hyperlipidemia, and smoking. Significance was accepted at *p* < 0.05. Also, we used analysis of variance (ANOVA) followed by Newman’s test to analyze continuous data. All of the examined polymorphisms were consistent with Hardy–Weinberg equilibrium (HWE) (*p* > 0.05). Furthermore, the HAPSTAT program (v.3.0, http://www.bios.unc.edu/*lin/hapstat/) was used to estimate the frequency of all allele combinations and to confirm those combinations with strong synergistic effects. Survival curves were created using the Cox proportional hazards regression, and the log-rank test was used to estimate the importance of differences between groups. Cox regression models were used to analyze the independent prognostic importance markers, and the results were adjusted for various factors, including sex, age, DM, HTN, hyperlipidemia, and smoking. Lastly, the statistical power of positive association was calculated using G*POWER 3.0 (http://www.psycho.uni-duesseldorf.de/abteilungen/aap/gpower3/).

## 3. Results

### 3.1. Clinical Profiles of Ischemic Stroke Patients and Controls

Table 1 and Table S1 show the baseline characteristics of the ischemic stroke and control groups. The male proportions of the control and the ischemic stroke groups were 42.7% and 46.2%, respectively. The mean ages of the ischemic stroke and control groups were 63.14 ± 11.63 and 62.70 ± 11.55 years, respectively. Significant differences in various clinical factors were identified between the ischemic stroke and control groups. 

### 3.2. Comparisons for the Four miRNA Polymorphisms among Patients with Ischemic Stroke, Ischemic Stroke Subtypes, and Controls

In Table 2 we present the logistic regression analysis, which demonstrated significant genotype frequency differences among ischemic stroke, LAD, SVD, and CE patients and the control group. Interestingly, the *miR-300* rs12894467 TC genotype and the dominant model (TT vs. TC + CC) were significantly associated with an increased risk for CE, which is an ischemic stroke subtype. Also, the statistical powers of positive association measured in this study were shown in Table S2 and these values of statistical power were 62.12% (*miR-300* rs12894467 dominant model) and 66.29% (*miR-300* rs12894467 TC genotype), respectively. Unfortunately, none of the other miRNAs examined were found to have significant associations with ischemic stroke or any subtypes. Furthermore, to determine whether other factors contributed to the association between genotype and ischemic stroke, we investigated the combined effects between miRNA polymorphisms and clinical factors by performing an interaction analysis to identify ischemic stroke prevalence. We identified synergistic effects between the four miRNA polymorphisms and various clinical factors using an interaction analysis (Tables S3 and S4, Figures S1 and S2). Next, we performed allele combination analyses among the four miRNA polymorphisms and compared their occurrences between the ischemic stroke and control groups (Table S5).

### 3.3. Analysis of the Four miRNA Polymorphisms, with Respect to Survival, in Ischemic Stroke Patients

**Figure 1:**
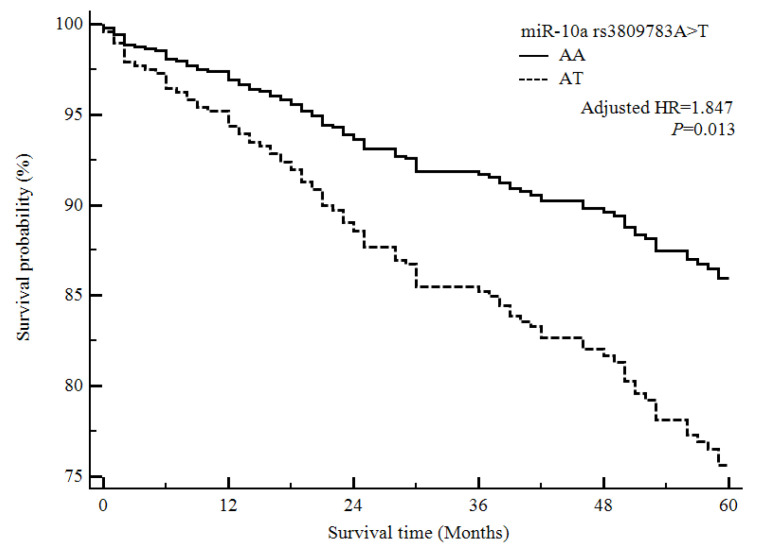
Survival analysis of ischemic stroke patients based on the *miR-10a* rs3809783 A>T polymorphism, using the Cox proportional hazards model. The survival probability of ischemic stroke patients in the *miR-10a* rs3809783 AA genotype is higher than that for ischemic stroke patients in the *miR-10a* rs3809783 AT genotype. Abbreviation: HR, hazard ratio.

**Figure 2:**
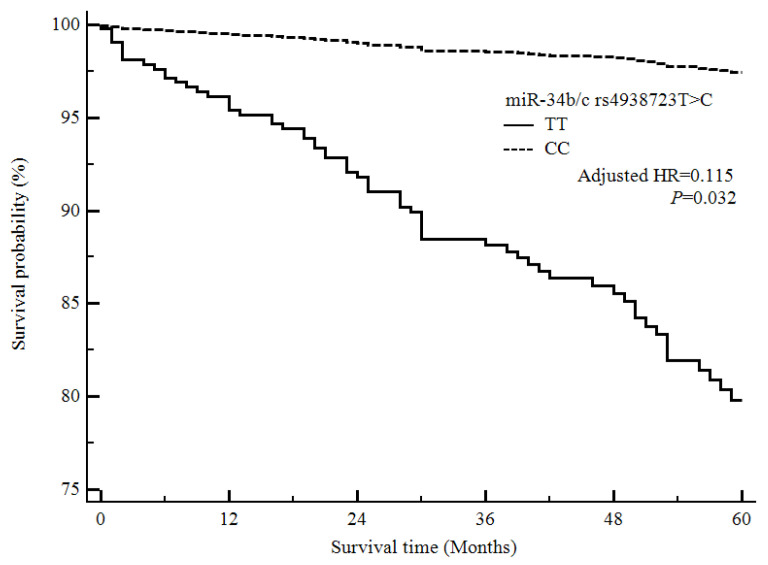
Survival analysis of ischemic stroke patients based on the *miR-34b/c* rs4938723 T>C polymorphism, using the Cox proportional hazards model. The survival probability of ischemic stroke patients in the *miR-34b/c* rs4938723 CC genotype is higher than that of ischemic stroke patients in the *miR-34b/c* rs4938723 TT genotype. Abbreviation: HR, hazard ratio.

Our final analysis investigated the association between the four miRNA polymorphisms and survival among ischemic stroke patients (Figure 1 and Figure 2). We performed a Cox proportional analysis, and the results showed that the *miR-10a* rs3809783 A>T and *miR-34b/c* rs4938723 T>C polymorphisms were associated with the five-year mortality rates among ischemic stroke patients. The *miR-10a* rs3809783 AT genotype and the dominant model (AA vs. AT + TT) were associated with decreased survival among ischemic stroke patients (Figure 1). Furthermore, the *miR-34b/c* rs4938723 CC genotype, the dominant model (TT vs. TC + CC), and the recessive model (TT + TC vs. CC) were associated with increased survival among ischemic stroke patients (Figure 2).

## 4. Discussion

Previously, miRNAs have been used as diagnostic markers for ischemic stroke and several other diseases, and the differential expression of miRNAs between diseased and normal conditions is known to affect disease progression [62]. Furthermore, miRNA functions have been reported to affect the expression of various target genes that are abnormally expressed in several diseases [6,52,63,64,65,66]. All of the miRNAs included in our study, *miR-10a*, *miR-27a*, *miR-34b/c*, and *miR*-*300*, were predicted to target either *PAI-1* or *ACE* mRNA, based on previous miRNA studies performed for ischemic stroke [6,18,48,49,50,67]. *PAI-1* is known to be involved in the thrombosis and fibrinolysis pathways, as a regulator of tissue plasminogen activator, and has been associated with thrombosis, organ failure, cancer, atherosclerosis, and tissue fibrosis [68,69,70,71,72,73,74,75]. *ACE* is associated with the thrombosis pathway, and several studies have demonstrated associations between *ACE* and *PAI-1* and between *ACE* and ischemic stroke [50,76,77]. The direct regulatory effects and predicted targeting effects against *ACE* and *PAI-1*, mediated by *miR-10a*, *miR*-*27a*, *miR*-*34b/c*, and *miR*-*300*, have been described by previous studies [43,44,45,46,66]. Moreover, we have already reported the association between another miRNA and ischemic stroke, and many studies in another population demonstrated the association between other miRNAs and ischemic stroke [78,79,80,81].

**Figure 3:**
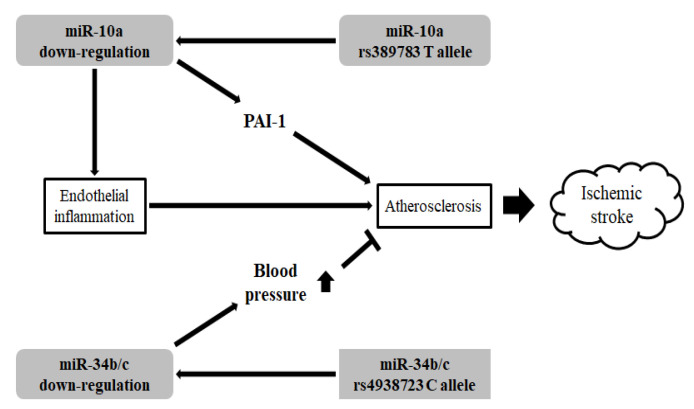
Schematic representation of the regulatory network of single nucleotide polymorphisms (SNPs) associated with miRNA. The potential impact of *miR-10a* and *34b/c* on atherosclerosis is the primary cause of ischemic stroke.

In our study, the *miR-300* rs12894467 TC genotype and the dominant model were associated with increased ischemic stroke susceptibility, and the *miR-10a* rs3809783 T allele was associated with increased ischemic stroke prevalence compared with the AA genotype when homocysteine levels exceeded 13.6 µmol/L. Furthermore, the *miR-27a* rs895819 C allele, when combined with either DM or homocysteine levels above 13.6 µmol/L, was associated with increased ischemic stroke prevalence compared with the TT genotype. In the Cox survival analysis, the *miR-10a* rs389783 AT genotype and the dominant model were associated with increased mortality among ischemic stroke patients, whereas the *miR-34b/c* rs4938723 CC genotype and the dominant model were associated with the decreased mortality of ischemic stroke patients. In Li et al., the *miR-10a* rs389783 T allele, compared with the A allele, showed decreased expression levels of the mature *miR-10a*, and it was connected that the *miR-10a* rs389783 T allele affects the down-regulation [55]. In Kumar et al. and Jiang et al., down-regulation or loss of *miR-10a* has promoted endothelial inflammation, and it is affected atherosclerosis that is the primary cause of ischemic stroke [16,82]. Also, in Wei et al., the *miR-34b/c* rs4938723 C allele, compared with the T allele, showed to decrease the transcriptional activity of the *miR-34b/c* promoter, and this study demonstrated that the *miR-34b/c* rs4938723 C allele affected the down-regulation [83]. In Yang et al., down-regulation of *miR-34b/c* promoted the elevation of blood pressure and affected atherosclerosis [84]. Therefore, *miR-10a* and *34b/c* polymorphism can affect expression levels of the miRNA themselves and affected miRNAs can show the different results of the expression levels of their target genes (Figure 3). Abnormal *PAI-1* and *ACE* expression levels, which are the target genes associated with the miRNAs examined in this study, may affect the thrombosis pathway and increase ischemic stroke risk (Figure 3). 

In the long term, these changes may affect the survival rate among ischemic stroke patients. Furthermore, miRNAs can be used for the early diagnosis and prediction of ischemic stroke risks. The synergistic effects between the expression of miRNA polymorphisms and major clinical factors can also increase the risk of ischemic stroke prevalence.

This study has several limitations that must be considered when interpreting our results. First, whether miRNA polymorphisms can be used to predict phenotypes associated with ischemic stroke prevalence remains unclear. Second, we performed our subgroup analyses on a limited pool of patients from the Korean population. Third, we did not perform a replication study between miRNA polymorphisms and other diseases associated with ischemic stroke. Therefore, even with the powerful evidence identified among these groups, the identification of causal effects is difficult. Additional studies, such as replication studies, are necessary to confirm whether any miRNA polymorphisms play crucial roles in ischemic stroke pathogenesis and to provide further evidence regarding whether the regulation of *PAI-1* and *ACE* expression can be used to prevent ischemic strokes. Although this was not a replication study, the recruitment of almost 1000 individuals from an ethnically homogeneous population (Koreans have a low degree of interracial marriage) is sufficient to provide reliable data. Therefore, our findings suggest that these polymorphisms may represent potential biomarkers that can be used to diagnose ischemic stroke and assess risk. 

## 5. Conclusions

In conclusion, we found that the *miR-300* polymorphism was associated with increased ischemic stroke susceptibility among the Korean population. Additionally, polymorphisms in *miR-10a* and *miR-34b/c* were associated with the increased or decreased mortality of ischemic stroke patients. This study marks the first report of an association between ischemic stroke and miRNA polymorphisms (*miR-10a*A>T, *miR*-*27a*T>C, *miR-34b/c*T>C, and *miR-300*T>C) in the Korean population. Additional studies of other racial and ethnic populations that examine the biological functions of miRNAs are necessary to fully understand the roles played by miRNA polymorphisms in ischemic stroke.

## Figures and Tables

**Table 1:** Baseline characteristics of ischemic stroke patients and controls.

Characteristics	Controls (*n* = 403)	Stroke Patients (*n* = 530)	*p*-Value	LAD Patients (*n* = 184)	*p*-Value	SVD Patients (*n* = 151)	*p*-Value	CE Patients (*n* = 56)	*p*-Value
Age (years, mean ± SD)	62.70 ± 11.55	63.14 ± 11.63	0.566	63.80 ± 11.16	0.281	60.88 ± 11.61	0.100	67.13 ± 12.65	0.008
Male (%)	172 (42.7)	245 (46.2)	0.281	82 (44.6)	0.669	76 (41.3)	0.107	24 (42.9)	0.980
Smoking (%)	137 (34.0)	208 (39.2)	0.083	71 (38.6)	0.241	56 (30.4)	0.464	18 (32.1)	0.784
Hypertension (%)	162 (40.2)	342 (64.5)	<0.0001	117 (63.6)	<0.0001	94 (51.1)	<0.0001	32 (57.1)	0.016
Hyperlipidemia (%)	81 (20.1)	161 (30.4)	0.0004	63 (34.2)	0.0002	47 (25.5)	0.006	12 (21.4)	0.817
Diabetes mellitus (%)	52 (12.9)	148 (27.9)	<0.0001	48 (26.1)	0.0001	45 (24.5)	<0.0001	14 (25.0)	0.016
MetS (%)	60 (14.9)	212 (40.0)	<0.0001	85 (46.2)	<0.0001	60 (32.6)	<0.0001	19 (33.9)	0.0004

*p*-values were calculated using a two-sided Student’s *t*-test for continuous variables and a chi-squared test for categorical variables; *p*-values were calculated by Mann–Whitney U test for continuous variables. Abbreviations: SVD, small-vessel disease; LAD, large-artery disease; CE, cardioembolism; SD, standard deviation; MetS, metabolic syndrome.

**Table 2:** Genotype frequency analyses of the four microRNA polymorphisms in stroke patients, categorized according to TOAST definitions, and controls.

Genotypes	Controls (*n* = 402)	Stroke Patients (*n* = 530)	AOR (95% CI)	*p*-Value	LAD (*n* = 184)	AOR (95% CI)	*p*-Value	SVD (*n* = 151)	AOR (95% CI)	*p*-Value	CE (*n* = 56)	AOR (95% CI)	*p*-Value
*miR-10a* rs3809783 A>T													
AA	340 (84.6)	435 (82.1)	1.000 (reference)		152 (82.6)	1.000 (reference)		121 (80.1)	1.000 (reference)		48 (85.7)	1.000 (reference)	
AT	58 (14.4)	89 (16.8)	1.126 (0.770–1.649)	0.540	30 (16.3)	1.107 (0.679–1.804)	0.685	26 (17.2)	1.256 (0.731–2.157)	0.409	8 (14.3)	1.008 (0.447–2.274)	0.984
TT	4 (1.0)	6 (1.1)	1.172 (0.307–4.481)	0.816	2 (1.1)	0.999 (0.176–5.663)	0.999	4 (2.6)	3.103 (0.678–14.204)	0.145	0 (0.0)	NA	0.994
Dominant (AA vs AT + TT)			1.129 (0.780–1.636)	0.520		1.103 (0.686–1.774)	0.686		1.370 (0.817–2.297)	0.232		0.949 (0.422–2.135)	0.900
Recessive (AA + AT vs TT)			1.153 (0.302–4.409)	0.835		1.045 (0.186–5.877)	0.961		2.962 (0.649–13.522)	0.161		NA	0.994
HWE-*P*	0.393	0.549											
*miR-27a* rs895819 T>C													
TT	203 (50.5)	254 (47.9)	1.000 (reference)		88 (47.8)	1.000 (reference)		73 (48.3)	1.000 (reference)		34 (60.7)	1.000 (reference)	
TC	158 (39.3)	223 (42.1)	1.145 (0.858–1.529)	0.358	80 (43.5)	0.963 (0.658–1.408)	0.844	63 (41.7)	1.135 (0.746–1.726)	0.555	17 (30.4)	0.653 (0.348–1.225)	0.184
CC	41 (10.2)	53 (10.0)	1.090 (0.678–1.753)	0.723	16 (8.7)	1.206 (0.670–2.172)	0.532	15 (9.9)	1.396 (0.698–2.793)	0.345	5 (8.9)	0.835 (0.301–2.316)	0.729
Dominant (TT vs TC + CC)			1.139 (0.866–1.499)	0.352		1.002 (0.703–1.429)	0.991		1.183 (0.796–1.759)	0.406		0.693 (0.387–1.240)	0.217
Recessive (TT + TC vs CC)			1.028 (0.654–1.616)	0.906		1.216 (0.699–2.115)	0.489		1.322 (0.685–2.549)	0.406		0.992 (0.367–2.680)	0.987
HWE-*P*	0.217	0.693											
*miR-34b/c* rs4938723 T>C													
TT	204 (50.7)	295 (55.7)	1.000 (reference)		100 (54.3)	1.000 (reference)		87 (57.6)	1.000 (reference)		27 (48.2)	1.000 (reference)	
TC	161 (40.0)	197 (37.2)	0.839 (0.627–1.123)	0.238	72 (39.1)	1.055 (0.728–1.527)	0.778	51 (33.8)	0.732 (0.476–1.125)	0.155	27 (48.2)	1.269 (0.706–2.281)	0.426
CC	37 (9.2)	38 (7.2)	0.703 (0.419–1.179)	0.182	12 (6.5)	0.852 (0.440–1.651)	0.636	13 (8.6)	0.883 (0.431–1.811)	0.734	2 (3.6)	0.374 (0.083–1.684)	0.200
Dominant (TT vs TC + CC)			0.816 (0.620–1.074)	0.147		1.008 (0.708–1.435)	0.964		0.766 (0.515–1.141)	0.190		1.080 (0.609-1.914)	0.793
Recessive (TT + TC vs CC)			0.762 (0.461–1.261)	0.291		0.816 (0.432–1.541)	0.531		1.022 (0.510–2.049)	0.952		0.310 (0.071–1.359)	0.120
HWE-*P*	0.522	0.518											
*miR-300* rs12894467 T>C													
TT	231 (57.5)	311 (58.7)	1.000 (reference)		114 (62.0)	1.000 (reference)		92 (60.9)	1.000 (reference)		23 (41.1)	1.000 (reference)	
TC	145 (36.1)	195 (36.8)	1.035 (0.774–1.384)	0.818	61 (33.2)	1.119 (0.771–1.625)	0.554	52 (34.4)	0.922 (0.605–1.404)	0.705	30 (53.6)	2.069 (1.141–3.753)	0.017
CC	26 (6.5)	24 (4.5)	0.729 (0.397–1.341)	0.309	9 (4.9)	0.820 (0.375–1.791)	0.618	7 (4.6)	0.733 (0.298–1.800)	0.498	3 (5.4)	1.175 (0.317–4.359)	0.809
Dominant (TT vs TC + CC)			0.986 (0.747–1.303)	0.923		1.070 (0.749–1.531)	0.709		0.898 (0.600–1.344)	0.600		1.931 (1.078–3.459)	0.027
Recessive (TT + TC vs CC)			0.720 (0.397–1.306)	0.280		0.782 (0.365–1.676)	0.527		0.758 (0.313–1.838)	0.540		0.823 (0.235–2.879)	0.760
HWE-*P*	0.615	0.343											

AOR was adjusted for age, gender, hypertension, diabetes mellitus, hyperlipidemia, and smoking status. Abbreviations: TOAST, Trial of ORG 10172 in Acute Stroke Treatment; AOR, adjusted odds ratio; CI, confidence interval; HWE, Hardy–Weinberg equilibrium; LAD, large-artery disease; SVD, small-vessel disease; CE, cardioembolism; HWE-P, Hardy-Weinberg Equilibrium *p*-value.

## Supplementary Materials

Supplementary materials can be found at https://www.mdpi.com/2075-1729/10/12/309/s1. Figure S1. The low density lipoprotein-cholesterol level was significantly different (P = 0.030) between miR-27a rs895819T allele (mean ± SD, 118.33 ± 34.16) and C allele (mean ± SD, 122.94 ± 39.28). Figure S2. The folate level was significantly different (P = 0.045) between miR-27a rs895819T allele (mean ± SD, 8.04 ± 7.40) and C allele (mean ± SD, 7.26 ± 4.98). Table S1. Baseline characteristics of ischemic stroke patients and controls. Table S2. Statistical power to detect various genetic associations in the present case-control study. Table S3. Ischemic stroke prevalence according to interaction analyses between the four miRNA genotypes and environmental factors. Table S4. Ischemic stroke prevalence by interaction analysis between four miRNA genotypes and environmental factors. Table S5. Allele combinations analysis among the four miRNA polymorphisms, in ischemic stroke patients and controls.
