# Supplementary material for: MiR-10a, 27a, 34b/c, and 300 Polymorphisms are Associated with Ischemic Stroke Susceptibility and Post-Stroke Mortality
Source: Life (Basel). 2020 Nov 25;10(12):309. doi: 10.3390/life10120309 (PMC7760023; doi:10.3390/life10120309)
Supplement: Supplementary file 1 [file life-10-00309-s001.pdf]

life

Supplementary Material of *MiR-10a, 27a, 34b/c*, and *300* Polymorphisms are Associated with Ischemic Stroke Susceptibility and Post-Stroke Mortality

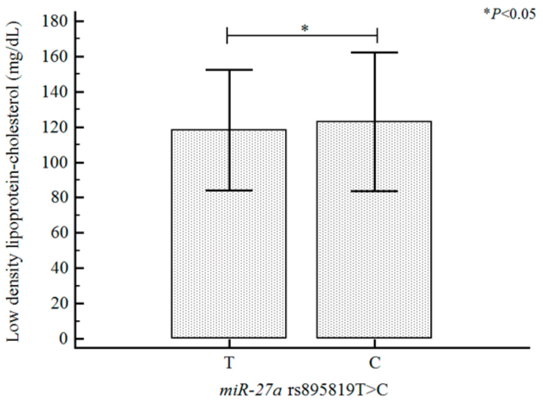

Figure S1. The low density lipoprotein-cholesterol level was significantly different (P = 0.030) between *miR-27a* rs895819T allele (mean ± SD, 118.33 ± 34.16) and C allele (mean ± SD, 122.94 ± 39.28).

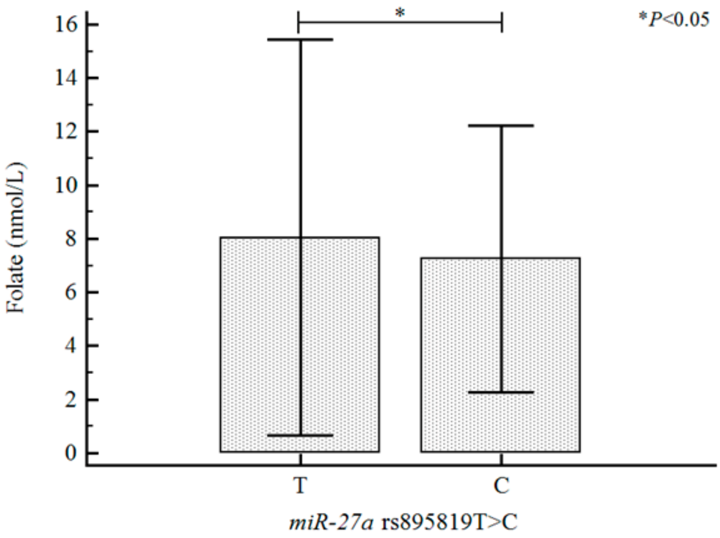

Figure S2. The folate level was significantly different (P = 0.045) between *miR-27a* rs895819T allele (mean ± SD, 8.04 ± 7.40) and C allele (mean ± SD, 7.26 ± 4.98).

life

MDPI

Table S1. Baseline characteristics of ischemic stroke patients and controls.

Characteristics	Controls (n = 403)	Stroke Patients (n = 530)	*P*-Value	LAD Patients (n = 184)	*P*-Value	SVD Patients (n = 151)	*P*-Value	CE Patients (n = 56)	*P*-Value
Triglyceride (mg/dL, mean ± SD)	144.12 ± 87.77	153.46 ± 113.16	0.337*	148.42 ± 86.91	0.584	170.06 ± 125.21	0.040*	136.57 ± 183.38	0.006*
FBS (mg/dL, mean ± SD)	114.29 ± 37.12	137.76 ± 59.42	<0.0001*	141.02 ± 63.61	<0.0001*	135.42 ± 54.47	<0.0001*	145.25 ± 67.23	<0.0001*
Total cholesterol (mg/dL, mean ± SD)	191.78 ± 37.14	190.21 ± 39.98	0.542	193.86 ± 46.90	0.934*	189.78 ± 36.37	0.572	179.89 ± 34.37	0.024
Platelet (10^3 cell/μL, mean ± SD)	243.75 ± 68.42	246.77 ± 87.68	0.510*	254.90 ± 87.32	0.694*	236.59 ± 66.98	0.272	244.91 ± 149.60	0.029*
PT (sec, mean ± SD)	11.77 ± 0.79	11.93 ± 3.25	0.749*	12.19 ± 5.26	0.612*	11.63 ± 0.78	0.092	12.10 ± 1.03	0.034*
aPTT (sec, mean ± SD)	33.59 ± 18.90	30.53 ± 4.51	0.065*	30.33 ± 4.76	0.024*	30.78 ± 4.58	0.304*	30.65 ± 4.02	0.666*
tHcy (μmol/L, mean ± SD)	9.97 ± 4.24	11.01 ± 6.29	0.001*	11.01 ± 5.07	0.004*	10.93 ± 5.46	0.011*	9.58 ± 4.32	0.522
Folate (nmol/L, mean ± SD)	8.80 ± 7.99	7.05 ± 5.55	<0.0001*	6.39 ± 4.15	<0.0001*	7.42 ± 6.31	0.0001*	8.61 ± 7.10	0.868
Creatinine (mg/dL, mean ± SD)	0.96 ± 0.24	1.03 ± 0.72	0.623*	1.01 ± 0.52	0.966*	1.08 ± 1.09	0.892*	0.99 ± 0.36	0.820*
Uric acid (mg/dL, mean ± SD)	4.67 ± 1.49	4.69 ± 1.56	0.861	4.67 ± 1.46	0.985	4.69 ± 1.53	0.855	4.63 ± 1.55	0.864
BUN (mg/dL, mean ± SD)	15.76 ± 4.90	16.25 ± 7.52	0.888*	15.34 ± 4.95	0.339	15.85 ± 9.55	0.118*	18.98 ± 10.77	0.018*
Fibrinogen (mg/dL, mean ± SD)	412.74 ± 136.98	425.56 ± 127.22	0.289	434.01 ± 130.31	0.154	396.40 ± 110.38	0.693*	454.56 ± 131.90	0.057

P-values were calculated using a two-sided Student's t-test, for continuous variables, and a chi-squared test, for categorical variables; * *P*-values were calculated by Mann-Whitney U test for continuous variables. Abbreviations: SVD, small-vessel disease; LAD, large-artery disease; CE, cardiogenic embolism; SD, standard deviation; FBS, fasting blood sugar; PT, prothrombin time; aPTT, activated partial thromboplastin time; tHcy, total homocysteine; BUN, blood urea nitrogen.

Table S2. Statistical power to detect various genetic associations in the present case-control study.

Characteristics	Table	AOR (95% CI)	Statistical Power (%)
miR-300 rs12894467 T>C TC genotype	Table 2 (CE)	2.069 (1.141–3.753)	66.29
miR-300 rs12894467 T>C CC genotype	Table 2 (CE)	1.175 (0.317–4.359)	4.92
miR-300 rs12894467 T>C dominant model	Table 2 (CE)	1.931 (1.078–3.459)	62.12
miR-300 rs12894467 T>C recessive model	Table 2 (CE)	0.823 (0.235–2.879)	2.83

Note: AOR, adjust odds ratio; 95% CI, 95% confidence interval.

Table 3. Ischemic stroke prevalence according to interaction analyses between the four miRNA genotypes and environmental factors.

Characteristic	*miR-10a* AA AOR (95% CI)	*miR-10a* AT+TT AOR (95% CI)	*miR-27a* TT AOR (95% CI)	*miR-27a* TC+CC AOR (95% CI)	*miR-34b/c* TT AOR (95% CI)	*miR-34b/c* TC+CC AOR (95% CI)	*miR-300* TT AOR (95% CI)	*miR-300* TC+CC AOR (95% CI)
Age (years)								
<63	1.000 (reference)	0.991 (0.564–1.740)	1.000 (reference)	1.194 (0.784–1.820)	1.000 (reference)	0.901 (0.591–1.373)	1.000 (reference)	1.281 (0.831–1.974)
≥63	0.744 (0.539–1.026)	0.896 (0.528–1.519)	0.798 (0.527–1.208)	0.811 (0.529–1.241)	0.747 (0.493–1.130)	**0.605 (0.393–0.931)**	0.862 (0.585–1.270)	0.661 (0.436–1.003)
Gender								
Male	1.000 (reference)	0.845 (0.488–1.463)	1.000 (reference)	1.209 (0.792–1.846)	1.000 (reference)	0.910 (0.596–1.390)	1.000 (reference)	0.926 (0.598–1.434)
Female	0.955 (0.666–1.368)	1.589 (0.882–2.862)	1.210 (0.752–1.948)	1.050 (0.653–1.688)	1.187 (0.737–1.912)	0.819 (0.520–1.292)	0.853 (0.547–1.331)	1.035 (0.649–1.650)
Hypertension								
No	1.000 (reference)	0.850 (0.493–1.465)	1.000 (reference)	0.931 (0.624–1.389)	1.000 (reference)	0.811 (0.542–1.212)	1.000 (reference)	1.159 (0.769–1.745)
Yes	**2.614 (1.922–3.556)**	**3.774 (2.150–6.625)**	**2.250 (1.511–3.351)**	**3.059 (2.037–4.595)**	2.732 (1.858–4.018)	2.257 (1.530–3.329)	**3.154 (2.166–4.593)**	**2.666 (1.770–4.013)**
Diabetes mellitus								
No	1.000 (reference)	0.971 (0.639–1.476)	1.000 (reference)	1.151 (0.848–1.563)	1.000 (reference)	0.897 (0.661–1.218)	1.000 (reference)	0.887 (0.650–1.209)
Yes	**1.940 (1.307–2.879)**	**4.173 (1.770–9.842)**	**2.297 (1.385–3.809)**	**2.580 (1.539–4.323)**	2.996 (1.791–5.012)	1.442 (0.864–2.407)	**1.697 (1.060–2.717)**	**2.862 (1.614–5.073)**
Hyperlipidemia								
No	1.000 (reference)	1.223 (0.798–1.874)	1.000 (reference)	1.195 (0.872–1.638)	1.000 (reference)	0.760 (0.555–1.043)	1.000 (reference)	0.983 (0.714–1.353)
Yes	1.798 (1.262–2.562)	1.619 (0.810–3.233)	**1.777 (1.100–2.871)**	**1.853 (1.195–2.873)**	1.455 (0.933–2.271)	1.438 (0.894–2.312)	1.754 (1.142–2.692)	1.711 (1.058–2.770)
Homocysteine (μmol/L)‡								
<13.6	1.000 (reference)	1.029 (0.689–1.535)	1.000 (reference)	1.095 (0.812–1.477)	1.000 (reference)	0.856 (0.634–1.155)	1.000 (reference)	1.029 (0.760–1.394)
≥13.6	1.413 (0.914–2.184)	**3.371 (1.082–10.503)**	1.519 (0.847 - 2.722)	**1.809 (1.019–3.213)**	2.134 (1.166–3.905)	1.112 (0.630–1.965)	1.781 (1.037–3.060)	1.342 (0.738–2.439)
Folate (nmol/L)†								
>3.47	1.000 (reference)	1.004 (0.666–1.514)	1.000 (reference)	1.134 (0.840–1.530)	1.000 (reference)	0.865 (0.640–1.169)	1.000 (reference)	1.036 (0.765–1.405)
≤3.47	**2.477 (1.522–4.031)**	**5.886 (1.939–17.861)**	3.132 (1.640 - 5.983)	3.025 (1.631–5.611)	3.849 (2.035–7.280)	2.115 (1.101–4.063)	4.048 (2.124–7.715)	2.151 (1.158–3.998)
Uric acid (mg/dL)‡								
<6.1	1.000 (reference)	1.378 (0.908–2.091)	1.000 (reference)	1.100 (0.812–1.489)	1.000 (reference)	0.918 (0.677–1.245)	1.000 (reference)	1.089 (0.800–1.483)
≥6.1	0.979 (0.627–1.528)	0.510 (0.218–1.193)	0.792 (0.447 - 1.403)	1.142 (0.640–2.036)	1.486 (0.811–2.723)	**0.553 (0.314–0.972)**	1.013 (0.595–1.723)	0.672 (0.369–1.223)

†Folate 3.47 nmol/L represents the bottom 15% cut-off value for ischemic stroke patients and controls.

‡Homocysteine 13.6 μmol/L and uric acid 6.1 mg/dL represent the upper 15% cut-off value for stroke patients and controls. The bolded AOR (95% CI) values indicate *P*-values lower than 0.05.

Table 4. Ischemic stroke prevalence by interaction analysis between four miRNA genotypes and environmental factors.

Characteristics	*miR-10a*AA AOR (95% CI)	*miR-10a*AT+TT AOR (95% CI)	*miR-27a*TT AOR (95% CI)	*miR-27a*TC+CC AOR (95% CI)	*miR-34b/c*TT AOR (95% CI)	*miR-34b/c*TC+CC AOR (95% CI)	*miR-300*TT AOR (95% CI)	*miR-300*TC+CC AOR (95% CI)
Smoking								
No	1.000 (reference)	1.344 (0.828 - 2.182)	1.000 (reference)	1.231 (0.872 - 1.738)	1.000 (reference)	0.805 (0.570 - 1.137)	1.000 (reference)	0.938 (0.663 - 1.327)
Yes	1.350 (0.940 - 1.937)	1.384 (0.743 - 2.578)	1.580 (0.969 - 2.576)	1.302 (0.824 - 2.056)	1.411 (0.878 - 2.267)	1.072 (0.671 - 1.713)	1.172 (0.756 - 1.819)	1.445 (0.887 - 2.353)
Total cholesterol (mg/dL)‡								
<200	1.000 (reference)	1.309 (0.814 - 2.105)	1.000 (reference)	1.088 (0.763 - 1.552)	1.000 (reference)	0.775 (0.543 - 1.104)	1.000 (reference)	1.093 (0.760 - 1.571)
≥200	0.905 (0.642 - 1.276)	0.890 (0.470 - 1.682)	0.740 (0.476 - 1.150)	1.069 (0.667 - 1.712)	0.835 (0.541 - 1.287)	**0.605 (0.379 - 0.964)**	0.891 (0.585 - 1.359)	0.866 (0.556 - 1.351)
Triglyceride (mg/dL)‡								
<150	1.000 (reference)	1.036 (0.646 - 1.661)	1.000 (reference)	1.074 (0.761 - 1.515)	1.000 (reference)	0.942 (0.668 - 1.328)	1.000 (reference)	0.990 (0.698 - 1.403)
≥150	0.914 (0.656 - 1.274)	1.295 (0.685 - 2.449)	0.838 (0.528 - 1.328)	1.121 (0.738 - 1.701)	1.278 (0.827 - 1.977)	0.739 (0.478 - 1.141)	0.983 (0.654 - 1.480)	1.021 (0.648 - 1.610)
HDL-cholesterol (mg/dL)								
≥40(M)/50(F)	1.000 (reference)	1.093 (0.546 - 2.190)	1.000 (reference)	1.488 (0.895 - 2.475)	1.000 (reference)	0.709 (0.429 - 1.172)	1.000 (reference)	1.199 (0.718 - 2.001)
<40(M)/50(F)	**1.726 (1.157 - 2.576)**	1.863 (0.909 - 3.818)	**1.965 (1.163 - 3.321)**	**2.234 (1.341 - 3.723)**	1.481 (0.889 - 2.470)	1.416 (0.821 - 2.445)	**2.145 (1.319 - 3.488)**	**1.808 (1.061 - 3.082)**
LDL-cholesterol (mg/dL)‡								
<130	1.000 (reference)	1.147 (0.639 - 2.059)	1.001 (reference)	1.419 (0.913 - 2.205)	1.003 (reference)	0.681 (0.438 - 1.057)	1.005 (reference)	1.025 (0.655 - 1.606)
≥130	1.285 (0.825 - 2.001)	1.314 (0.553 - 3.123)	1.306 (0.734 - 2.326)	1.488 (0.840 - 2.634)	1.074 (0.618 - 1.867)	1.040 (0.565 - 1.914)	1.287 (0.744 - 2.228)	1.208 (0.680 - 2.145)
PLT (10^3/uL)‡								
<306	1.000 (reference)	0.955 (0.632 - 1.443)	1.000 (reference)	1.098 (0.813 - 1.483)	1.000 (reference)	0.774 (0.573 - 1.047)	1.000 (reference)	1.027 (0.756 - 1.394)
≥306	0.783 (0.507 - 1.210)	2.002 (0.846 - 4.737)	0.820 (0.459 - 1.465)	1.096 (0.645 - 1.861)	0.819 (0.470 - 1.426)	0.855 (0.490 - 1.490)	1.039 (0.599 - 1.802)	0.873 (0.504 - 1.513)
PT (sec)‡								
<12.5	1.000 (reference)	1.048 (0.670 - 1.640)	1.000 (reference)	1.321 (0.948 - 1.840)	1.000 (reference)	0.792 (0.568 - 1.102)	1.000 (reference)	0.983 (0.702 - 1.377)
≥12.5	1.059 (0.680 - 1.652)	1.695 (0.659 - 4.362)	**1.885 (1.026 - 3.465)**	0.983 (0.560 - 1.724)	1.139 (0.649 - 1.998)	0.871 (0.477 - 1.588)	1.048 (0.604 - 1.817)	1.157 (0.634 - 2.110)
aPTT (sec)‡								
<35.3	1.000 (reference)	0.983 (0.634 - 1.523)	1.000 (reference)	1.133 (0.812 - 1.581)	1.000 (reference)	0.835 (0.598 - 1.165)	1.000 (reference)	1.035 (0.739 - 1.450)
≥35.3	**0.521 (0.335 - 0.809)**	1.702 (0.506 - 5.727)	**0.532 (0.289 - 0.978)**	0.766 (0.436 - 1.346)	0.684 (0.398 - 1.177)	**0.402 (0.208 - 0.777)**	0.647 (0.379 - 1.103)	**0.518 (0.275 - 0.975)**
Fibrinogen (mg/dL)‡								
<541	1.000 (reference)	0.976 (0.567 - 1.682)	1.000 (reference)	1.136 (0.753 - 1.712)	1.000 (reference)	0.804 (0.533 - 1.214)	1.000 (reference)	1.095 (0.719 - 1.668)
≥541	0.983 (0.548 - 1.763)	1.480 (0.313 - 6.993)	0.888 (0.410 - 1.925)	1.317 (0.591 - 2.935)	0.721 (0.345 - 1.507)	1.200 (0.523 - 2.755)	1.326 (0.599 - 2.935)	0.871 (0.408 - 1.859)
BUN (mg/dL)‡								
<20.8	1.000 (reference)	1.218 (0.816 - 1.817)	1.000 (reference)	1.195 (0.887 - 1.612)	1.000 (reference)	0.746 (0.552 - 1.006)	1.000 (reference)	1.085 (0.800 - 1.472)
≥20.8	1.316 (0.859 - 2.015)	1.166 (0.412 - 3.296)	1.482 (0.848 - 2.588)	1.215 (0.688 - 2.145)	0.929 (0.540 - 1.596)	1.264 (0.694 - 2.302)	1.586 (0.894 - 2.812)	1.036 (0.602 - 1.782)

Abbreviation: HDL, high density lipoprotein; LDL, low density lipoprotein; PLT, platelet; PT, prothrombin time; aPTT, activated partial thromboplastin time; BUN, blood urea nitrogen

‡Total cholesterol 200 mg/dL, Triglyceride 150 mg/dL, LDL-cholesterol 130 mg/dL, PLT 306 $10^3/\mu\ell$, PT 12.5 sec, and aPTT 35.3 sec, Fibrinogen 541 mg/dL, BUN 20.8 mg/dL were upper 15% cut-off each level in stroke patients and controls. The bold of AOR (95% CI) has the *P*-value and these *P*-value was lower than 0.05.

life

MDPI

Table S5. Allele combinations analysis among the four miRNA polymorphisms, in ischemic stroke patients and controls.

Allele Combinations	Controls (2n = 804)	Stroke Patients (2n = 1,060)	OR (95% CI)	*P*-Value†
miR-10a A>T/*miR-27a* T>C/*miR-34b/c* T>C/*miR-300* T>C				
A-T-T-T	283 (35.3)	368 (34.8)	1.000 (reference)	
A-T-C-C	41 (5.1)	32 (3.1)	0.600 (0.369–0.978)	0.039
A-C-T-C	40 (5.0)	32 (3.0)	0.615 (0.377–1.004)	0.050
T-T-T-C	4 (0.5)	20 (1.8)	3.845 (1.299–11.380)	0.010††
T-T-C-T	1 (0.2)	10 (1.0)	7.690 (0.978–60.460)	0.029††
T-T-C-C	12 (1.5)	0 (0.0)	0.031 (0.002–0.522)	<0.0001††
T-C-T-C	4 (0.5)	18 (1.7)	3.461 (1.158–10.340)	0.026††
miR-10a A>T/*miR-27a* T>C/*miR-300* T>C				
A-T-T	396 (49.2)	513 (48.4)	1.000 (reference)	
T-C-C	1 (0.1)	18 (1.7)	13.890 (1.846–104.600)	0.001
miR-10a A>T/*miR-34b/c* T>C/*miR-300* T>C				
A-T-T	399 (49.7)	552 (52.1)	1.000 (reference)	
T-T-C	8 (1.0)	37 (3.5)	3.343 (1.540–7.258)	0.001
T-C-C	10 (1.2)	0 (0.0)	0.034 (0.002–0.590)	0.0002††
miR-27a T>C/*miR-34b/c* T>C/*miR-300* T>C				
T-T-T	308 (38.3)	405 (38.2)	1.000 (reference)	
T-C-C	51 (6.3)	31 (3.0)	0.462 (0.289–0.740)	0.001
miR-34b/c T>C/*miR-300* T>C				
T-T	439 (54.5)	599 (56.6)	1.000 (reference)	
C-C	66 (8.3)	55 (5.2)	0.611 (0.418–0.892)	0.010

OR, odds ratio; 95% CI, 95% confidence interval; N/A, not applicable; †chi-squared test; †† Fisher's exact test. *P*-value >0.1 were excluded for Table 3.

Publisher's Note: MDPI stays neutral with regard to jurisdictional claims in published maps and institutional affiliations.

© 2020 by the authors. Submitted for possible open access publication under the terms and conditions of the Creative Commons Attribution (CC BY) license (http://creativecommons.org/licenses/by/4.0/).
